# Fingertip cutaneous metastasis of salivary duct carcinoma secondary to scald: A unique case report and a brief review of literature (following CARE guidelines)

**DOI:** 10.1097/MD.0000000000038965

**Published:** 2024-07-19

**Authors:** Ran Peng, Wenqi Wu, Xuemin Li, Yang Zhou, Jing Su, Hao Wang

**Affiliations:** a Department of Radiation Oncology, Peking University Third Hospital, Beijing, People’s Republic of China; b Peking University Health Science Center, Beijing, People’s Republic of China; c Department of Radiation Oncology, Da Qing Long Nan Hospital, Daqing City, Heilongjiang Province, People’s Republic of China; d Department of Pathology, Peking University Third Hospital, Beijing, People’s Republic of China; e Cancer Center, Peking University Third Hospital, Beijing, People’s Republic of China.

**Keywords:** case report, cutaneous metastasis, radiotherapy, salivary duct cancer, scald

## Abstract

**Rationale:**

Salivary duct carcinoma (SDC) is an aggressive form of cancer, with cutaneous metastasis being a rare occurrence. Furthermore, cutaneous metastasis of SDC secondary to a scald is even rarer, and to the best of our knowledge, our case represents the first such instance. Considering the involvement of the fingers in the metastatic site, which may affect limb function and quality of life, we present this case to explore the reason why scald could lead to distant recurrence and better treatment options.

**Patient concerns:**

An 85-year-old man diagnosed with SDC in the parotid gland found enlarged masses at the fingertips as a consequence of a burn, 6 years after his initial treatment.

**Diagnoses:**

Cutaneous metastasis of SDC in the parotid gland and left thumb loss due to surgery.

**Interventions:**

Radiotherapy was offered, targeting at the masses on the fingers, with dose at 15 Gy in 3 fractions, 12 Gy in 3 fractions, 15 Gy in 3 fractions for both hands and additional 21 Gy in 7 fractions only for left hand.

**Outcomes:**

The tumors shrank after 2 months of radiotherapy and the patient recovered well. Side effects included nail hyperplasia and paronychia.

**Lessons:**

Connections between scald and distant metastasis of malignant tumors in this case needed further investigation. Considering reserving function of the fingers while dealing with metastasis, radiotherapy is recommended rather than surgery.

## 1. Introduction

Salivary duct carcinoma (SDC) is a rare and highly aggressive salivary gland malignancy with a poor prognosis. SDC typically metastasizes to local lymph nodes, lungs, or the brain.^[1]^ In this report, we present a unique case of a patient who developed metastases on the fingertip skin of both hands following scald injuries to his fingers. Fingertip metastasis of SDC has never been reported before, making this the only known case. According to the literature, only 8 cases of skin metastasis of SDC have been reported worldwide.^[2–7]^ In order to improve the understanding of this condition and its treatment, we report on this exceptional 85-year-old patient. This study has been approved by Peking University Third Hospital Medical Science Research Ethics Committee.

## 2. Case report

### 2.1. Patient information

An 85-year-old male was diagnosed with primary ductal carcinoma of the parotid gland. He underwent a right parotid gland mass resection in May 2014 and tumor bed radioactive seed implantation in August of the same year.

### 2.2. Clinical findings

**Figure 1.**
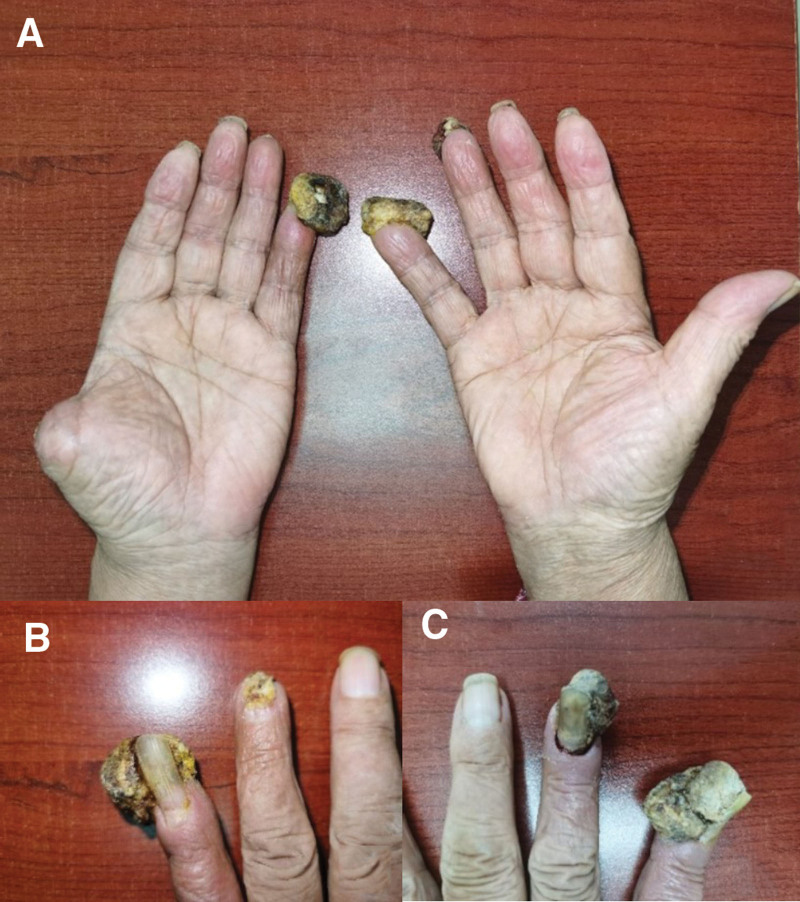
Photos showing the hands with resting metastasis after finger resection. A, B and C show different angles.

**Figure 2.**
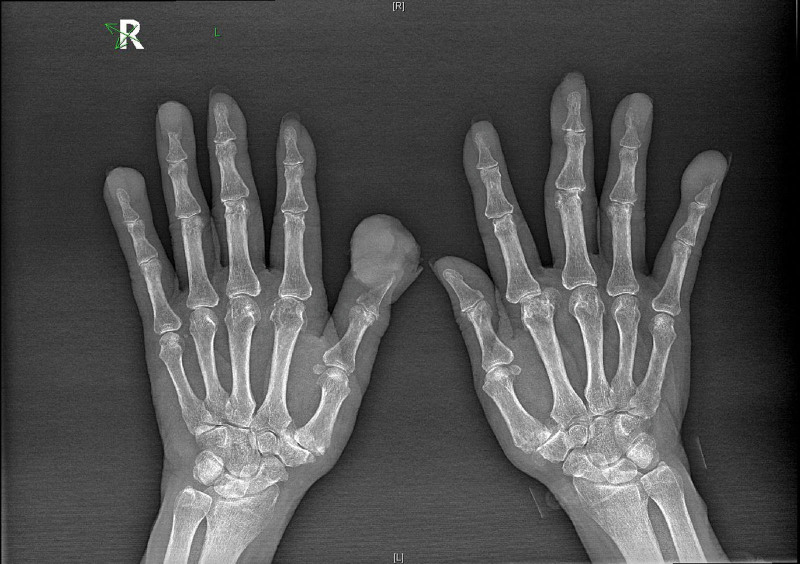
X-rays of both hands before finger resection showing metastatic lesions.

In November 2019, he was admitted to the hospital, where a neck mass was discovered on the left side. Local radioactive seed implantation was performed in May 2020. In June 2020, the patient scalded all 10 of his fingers, and gray masses began to grow, progressively enlarging and ulcerating on the surface of the left 1st, 4th, and 5th fingers, as well as the right 3rd, 4th, and 5th fingers. The largest mass measured 4 × 3.5 × 2.5 cm in size (see Figs. 1 and 2).

### 2.3. Timeline (see Table 1)

### 2.4. Diagnostic assessment

**Figure 3.**
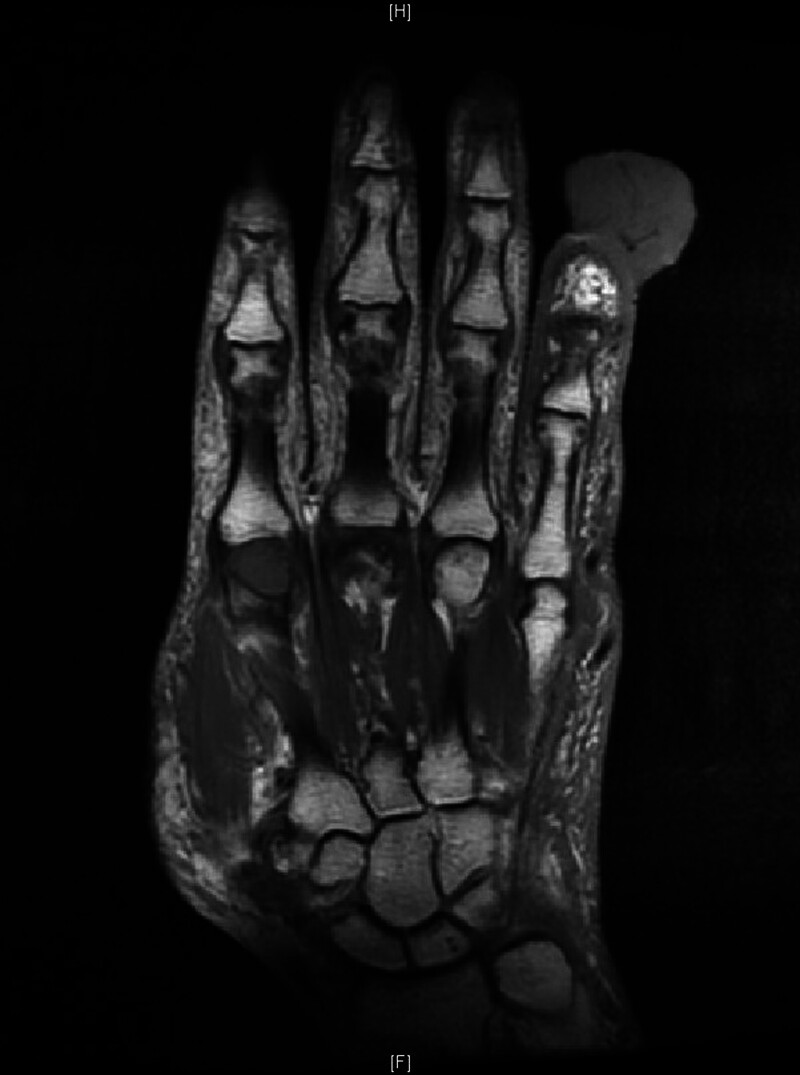
MRI of the left hand after finger resection showing resting metastatic lesions.

On August 6, 2020, a biopsy of the left-hand finger revealed poorly differentiated carcinoma infiltration in the skin tissue and the presence of intravascular cancer emboli. Consequently, a left thumb amputation was performed, which significantly impacted the function of the patient left hand. As a result, the patient refused any further surgical interventions (see Fig. 3).

**Table 1:** Timeline of diagnosis and treatment.

2014.5	Diagnosed with primary ductal carcinoma of the parotid gland; Right parotid gland mass resection.
2014.8	Tumor bed radioactive seed implantation.
2019.11	A neck mass was discovered on the left side.
2020.5	Local radioactive seed implantation.
2020.6	Fingers were scalded and mass was founded on fingers.
2020.8	Multi-finger biopsy of both hands. Left thumb amputation.
2020.10	Radiotherapy.
2020.10~2022.6	Brixine intake. (Ubenimex Ubenmax capsules).

**Figure 4.**
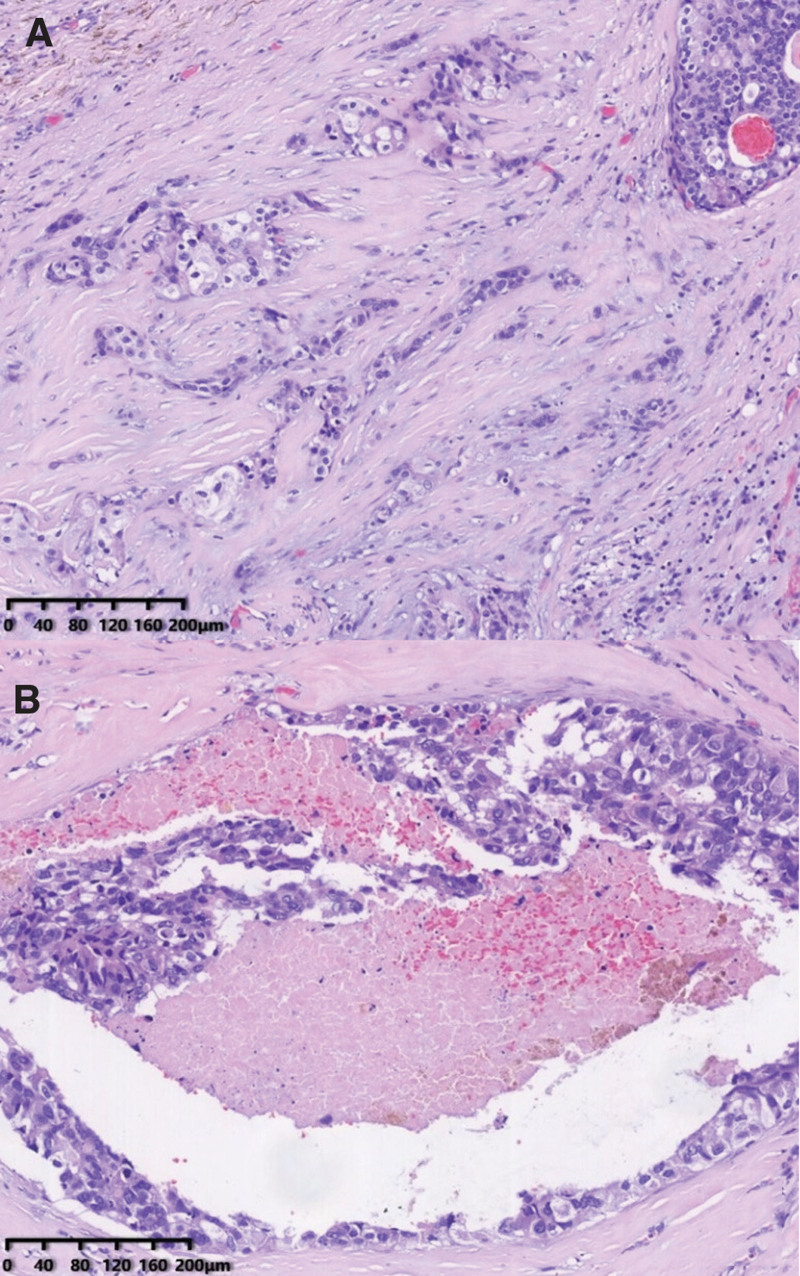
Section from the parotid gland showing cells with marked atypia grew infiltratively in the stroma and structure resembling high-grade ductal carcinoma in situ of the breast and necrosis in the center of the tumor cluster.

**Figure 5.**
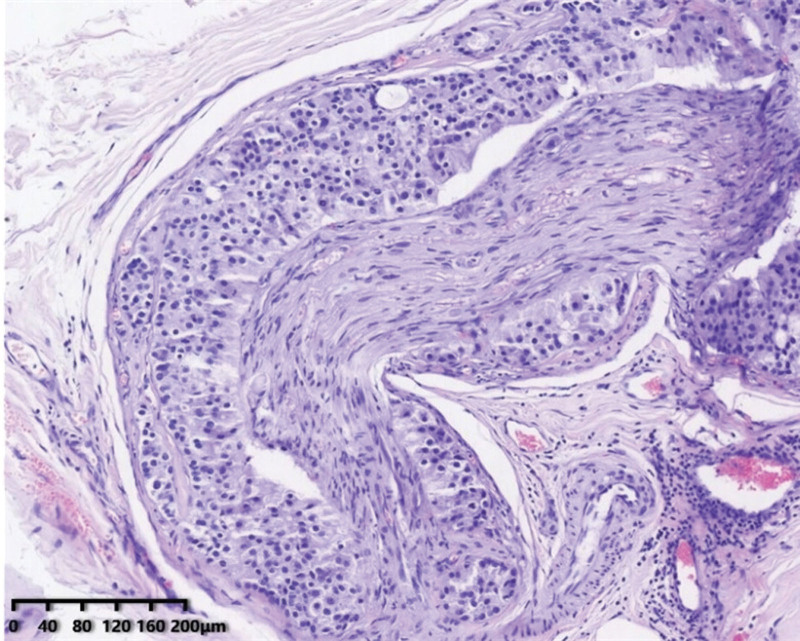
Section of the parotid gland showing perineural invasion.

**Figure 6.**
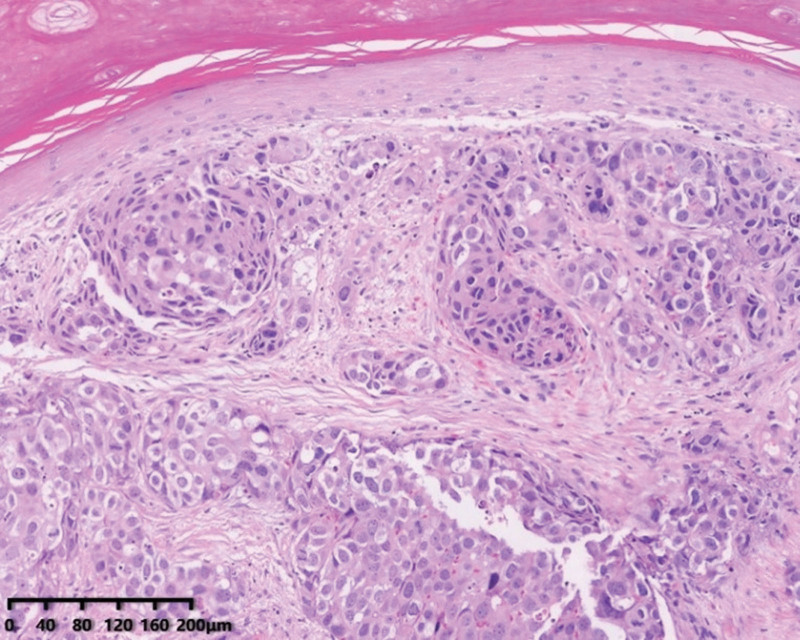
Section from the fingertip neoplasms showing the adenocarcinoma with invasive growth in the dermis and no perineural invasion.

On August 31, 2020, the patient underwent a multi-finger biopsy of both hands. Sections of the surface of the left 4th and 5th fingers, as well as the right 3rd, 4th, and 5th fingers, revealed low-differentiated carcinoma infiltration and intravascular cancer thrombi. The primary lesions were predominantly cystic with invasive growth, and the invasive characteristics of the fingertip metastases were similar to those of the primary lesions. However, it is worth noting that multiple nerve invasions were observed in the primary tumor, but no nerve invasion was detected in the metastatic tumor (see Figs. 4, 5, and 6).

**Figure 7.**
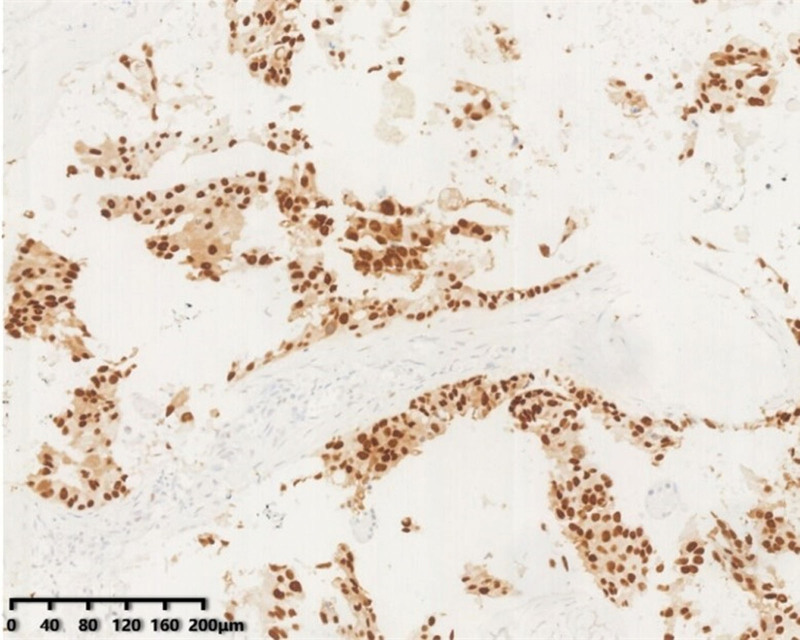
AR staining of fingertip neoplasms showing AR-positive.

**Figure 8.**
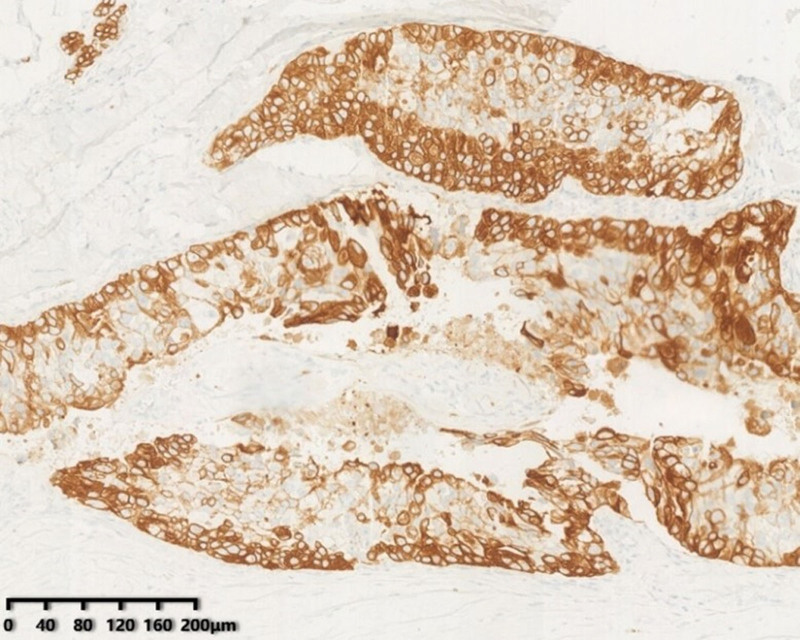
CK7 staining of fingertip neoplasms showing CK7 positive.

HER2 immunohistochemical result was negative. Moreover, gene testing results indicated that the mutation and abundance of ERBB2 exon > 20%; NTRK1 SNV is higher; TP53 mutations. Additionally, the tumor cells expressed AR, frequently expressed in salivary duct cancer and CK7, the adenocarcinoma marker (see Figs. 7 and 8).

### 2.5. Therapeutic intervention

Radiotherapy for the remaining affected fingers was offered to the patient. Left fingertip radiotherapy commenced in October 2020. The target area was defined as the masses on the left ring finger and little finger. The prescribed dose was 15 Gy in 3 fractions, followed by 12 Gy in 3 fractions and 15 Gy in 3 fractions. Due to a less-than-optimal response to the radiation, an additional dose of 21 Gy in 7 fractions was administered to the little finger. After a month of treatment, the lesion on the patient left finger significantly reduced in size and formed a shell, which fell off layer by layer during subsequent follow-ups. The right fingers also received radiotherapy with the same prescribed dose as the left, without an additional dose.

From October 20, 2020 to June 23, 2022, the patient continued taking Brixine (Ubenimex Ubenmax capsules).

### 2.6. Follow-up and outcomes

**Figure 9.**
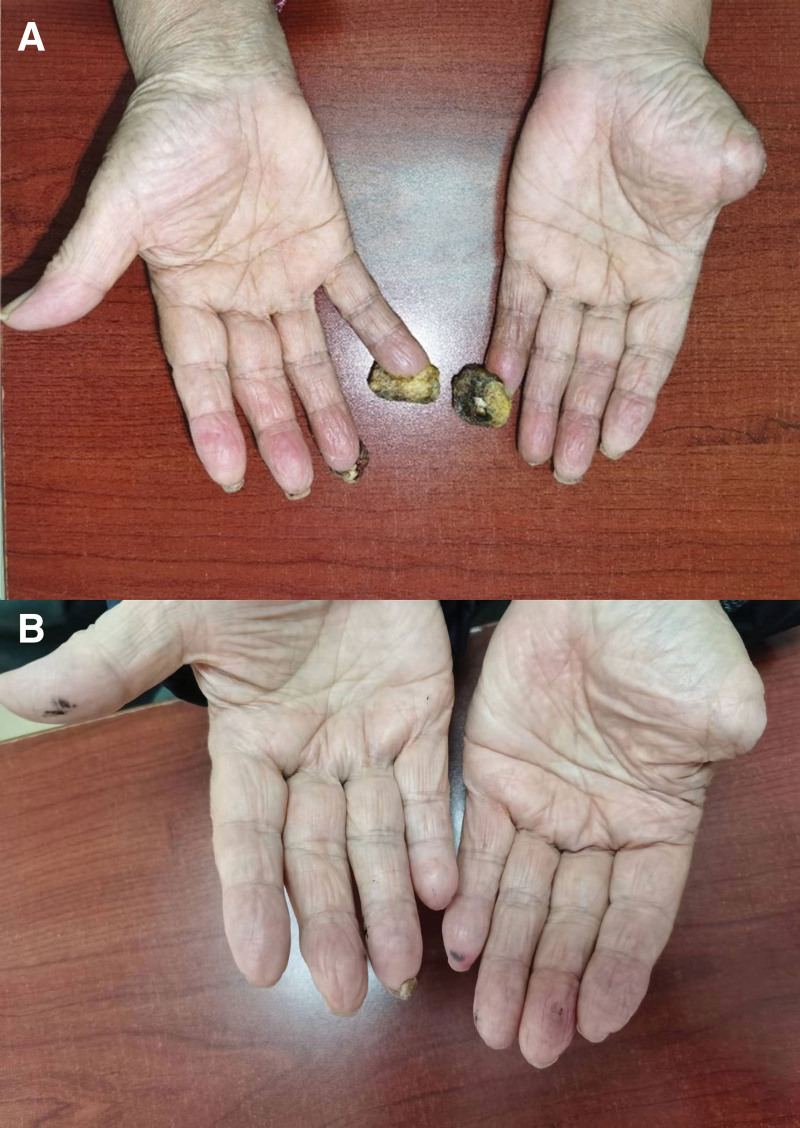
Photos showing the recovery of the fingers (A by 2020.10, and B by 2021.4).

By June 2022, the tumors had gradually shrunk after 2 months of radiotherapy. However, due to the excision of his left thumb, the patient experienced a loss of function in his left hand. Additionally, nail hyperplasia occurred in the radiotherapy area of the left 4th and 5th fingers, as well as the right 3rd, 4th, and 5th fingers. The most severe side effect was paronychia. After receiving appropriate treatment, the patient condition improved, and he was in good health at the time of publication (see Fig. 9)

## 3. Discussion

SDC is a highly aggressive subtype of salivary gland carcinoma (SGC), accounting for 4% to 10% of all SGC cases. It typically occurs in the parotid gland and is prone to recurrence and distant metastasis, resulting in a low overall survival rate.^[8]^ However, cutaneous metastasis is a rare occurrence.^[9]^ To the best of our knowledge, there have only been 8 reported cases of SDC with cutaneous metastasis in the literature^[2–7,10]^ (see Table 2). Among these cases, 2 involved metastasis to a large area of the anterior chest skin, 1 case to the anterior ear skin, 2 cases exhibited prominent vascular components, and 3 cases presented with nodules. Only 1 case was found in the submandibular gland10. If we broaden our scope to include extremities, there has been only one reported case of skin metastases of SDC, which was located at the roots of the arms.^[6]^

**Table 2:** Comparison of cases.

Case	Age	Gender	Original site	Dose of radiotherapy	Site/onset age of cutaneous metastasis	Nerve invasion of original site	Lymph node involvement
Chakari, 2017^[10]^	78	Male	The posterior pole of the submandibular gland on the right side	70Gy	The right side of the forehead/79	Perineural invasion	Positive
Aygit, 2005^[6]^	40	Male	Left parotid gland	60Gy	Left scapular area and the left suprapatellar region/41	No	Nm
Case 1 of Cohen, 2012^[5]^	71	Male	Left parotid gland	60Gy	Chest, neck, and posterior shoulder/73	No	Infiltration of lymph vessels
Case 2 of Cohen, 2012^[5]^	69	Male	Left parotid gland	45Gy	Left axilla and chest wall/69	NM	Positive
Zanca, 1993^[4]^	76	Female	Right parotid gland	NM	Right cheek and periauricular area/76	NM	Positive
Giltman, 1977^[3]^	23	Male	Right parotid gland	NM	Right parasagittal post operational region and right parotid region/58	Neuroma	Positive
Mirmohammad, 2020^[7]^	56	Male	Right parotid gland	NM	Right parotid region of skin/56	NM	Positive
Current report	85	Male	Right parotid gland	123Gy (for metastasis)	Skin of fingertips/85	Nerve invasion	Positive

NM = not mentioned.

Clinically, most SDC patients present with rapidly expanding lesions accompanied by facial palsy or pain. The rates of local recurrence, lymph node metastasis, and distant metastasis for SDC are 30%, 60%, and 30% to 70%, respectively.^[11]^ The most common sites of metastasis are the lungs (54%), bones (46%), and lymph nodes (42%), with brain metastases occurring in 18% of patients.^[1]^ Jaehne et al reported that the primary mode of distant metastasis for SDC is hematogenous metastasis.^[12]^

In general, cutaneous metastases of internal cancers are rare.^[13]^ In very rare cases, cutaneous metastases may be the first manifestation of a silent occult cancer, which often presents as a benign disease. When cutaneous metastases occur on the fingers, the most common cause is typically lung cancer.^[14]^

The main treatment methods for parotid ductal carcinoma include enlarged local excision with functional or radical neck dissection, supplemented by postoperative radiotherapy and chemotherapy.^[15]^ Although cutaneous metastasis typically requires local treatment such as resection, which may provide a high local control rate, simply amputating a thumb, in this case, could be a quick resolution for the tumor but a significant detriment to the patient quality of life. In contrast, the right hand, which received radiotherapy, recovered well both in terms of symptoms and function. This suggests that radiotherapy can provide good local control without compromising function for cutaneous metastases of SDC. Radiotherapy may be a suitable alternative when surgical tumor removal leads to significant functional loss.

The implications of scald injuries on cancer metastasis and related malignancies warrant in-depth investigation. There may be a potential correlation between benign hypertrophic scarring following burns and malignant transformation. A case study involving a 61-year-old Caucasian male, who experienced scalding at the age of 4, underwent radiation therapy for post-burn hypertrophic scarring, and subsequently developed multiple Marjolin ulcers on his left arm, chest, and right temporal scalp.^[16]^ Notably, while the Marjolin ulcer presented as squamous cell carcinoma pathologically, the current case was identified as ductal adenocarcinoma, exhibiting significant dissimilarity. We hypothesize that scald injuries may alter the microenvironment, promote the formation of blood vessels in the affected area, and compromise local immune function. Furthermore, scald injuries may trigger the release of inflammatory mediators. Increased expression of VEGF, PCNA, and vimentin was observed in the tumor tissue. The reduction of E-cadherin levels might contribute to changes in tumor tissue growth and metastasis.^[17]^ Consequently, these factors render the site more susceptible to the implantation of hematogenous or lymphogenous tumor cells.^[18]^

SDC typically exhibits an aggressive clinical course and unfavorable prognosis, with over 70% of patients succumbing within 3 years of initial diagnosis.^[19]^ Notably, the overexpression of HER2/neu and p53 has been identified as an adverse prognostic factor for early regional recurrence, distant metastasis, and diminished overall survival.^[20]^ Hassan et al reported a case involving delayed metastatic skin lesions at the primary site following total parotid gland excision, wherein the patient survived for over 6 years after the initial diagnosis before eventually succumbing to visceral metastases and associated complications.^[7]^ In the absence of visceral metastases, patients with SDC presenting solely with cutaneous metastases may experience a relatively more favorable prognosis.

There may still be some possible limitations in this study. First, this patient has been treated in several hospitals thus there could be partial loss of case information due to different ways of medical record or quality control. Second, although there is some literature on this kind of disease, there is still a lack of reference for this rare condition in the process of diagnosis, treatment and research, which may lead to an imperfect discussion.

In conclusion, this study contributes to the understanding of SDC and its metastatic behavior, suggesting that radiotherapy may be an effective alternative for managing cutaneous metastases without compromising function, sheds light on a rare case of SDC with unique metastatic patterns, highlighting the need for further research and comprehensive management strategies to improve outcomes for patients with this aggressive malignancy.

## Author contributions

**Conceptualization:** Ran Peng.

**Data curation:** Wenqi Wu.

**Formal analysis:** Xuemin Li.

**Investigation:** Ran Peng, Yang Zhou.

**Methodology:** Ran Peng.

**Resources:** Jing Su.

**Supervision:** Hao Wang.

**Writing – review & editing:** Ran Peng, Hao Wang.

**Writing – original draft:** Wenqi Wu.
